# Transiliac–Transsacral Screw Provides Good Outcomes for Stabilizing Unstable Fragility Fracture of the Pelvis: A Retrospective Case Series

**DOI:** 10.3390/life16010102

**Published:** 2026-01-11

**Authors:** Ping-Ying Yu, Kai-Cheng Lin, Yih-Wen Tarng, Chien-Jen Hsu

**Affiliations:** 1 Department of Orthopedics, Kaohsiung Veterans General Hospital, Kaohsiung City 813, Taiwan; pyyu0904@vghks.gov.tw (P.-Y.Y.);; 2 Department of Occupational Therapy, Shu-Zen Junior College of Medicine and Management, Kaohsiung City 821, Taiwan; 3 Department of Medical Education and Research, Kaohsiung Veterans General Hospital, Kaohsiung City 813, Taiwan

**Keywords:** fragility fracture, pelvis, osteoporosis

## Abstract

(1) Background: Fragility fractures of the pelvis (FFP) in elderly patients pose significant clinical challenges due to osteoporosis and associated morbidity. Transiliac–transsacral (TITS) screw fixation offers biomechanical advantages for stabilizing unstable posterior pelvic ring injuries, yet clinical outcomes remain underreported. We aim to report radiographic and clinical outcomes of TITS fixation for posterior pelvic ring injuries in FFP. (2) Methods: We conducted a retrospective review of 22 elderly female patients (mean age 79.0 ± 7.9 years) who underwent TITS screw fixation for unstable posterior pelvic ring fragility fractures between 2019 and 2024. Perioperative, radiographic, and functional outcomes were analyzed. (3) Results: Median operative time was 74 min (IQR 55–90 min), with minimal blood loss (median 5 mL). No intraoperative neurovascular injuries occurred. Median hospital stay was 7 days (IQR 5–10 days). At a mean follow-up of 6 months, 81.8% of patients maintained excellent or good reduction. Screw loosening was observed in 18.2% of cases, with only one (4.5%) requiring revision. Median VAS scores (range 0–10) decreased significantly from 5 preoperatively to 2 at discharge (*p* < 0.001). By discharge, 59.1% of patients were able to ambulate with assistance. (4) Conclusion: TITS screw fixation is a safe and feasible option for stabilizing unstable FFP in elderly, osteoporotic patients. It provides reliable mechanical stability, promotes early mobilization, and is associated with a short hospital stay and low complication rates.

## 1. Introduction

Fragility fractures of the pelvis (FFP) predominantly affect elderly patients, often resulting from low-energy trauma such as falls from a standing height. These fractures are closely associated with osteoporosis, in which decreased bone mineral density compromises the structural integrity of the pelvic ring. As the geriatric population continues to grow, FFP has become a significant clinical challenge.

The Rommens classification categorizes FFP into four types based on the stability of the pelvic ring and location of the instability, guiding treatment strategies that range from conservative management for stable fractures to surgical intervention for unstable ones (type IV, type III, and some of the type II cases that present progressive displacement) [1,2]. Type I fractures, characterized by slight instability in anterior pelvis, are managed conservatively with pain control and early mobilization. For type II fractures, characterized by nondisplaced posterior pelvic ring fractures, conservative treatment should be attempted first, while operative treatments are considered when conservative treatment fails. FFP type III, characterized by unilateral displacement of the posterior pelvis with associated anterior pelvic ring fracture, are associated with high degree of instability. Percutaneous fixation is favored for minimally displaced type III fractures, whereas open reduction and internal fixation remain an option for displaced injuries. Type IV fractures are described as bilateral displaced posterior pelvic ring fractures. They should be stabilized operatively like type III fractures, but with bilateral fixation of the pelvic ring [3].

Despite the widespread adoption of the Rommens classification as a framework for guiding treatment strategies, the optimal management of FFP remains controversial for several fracture subtypes. A recent systematic review and meta-analysis demonstrated that fragility fractures of the pelvis are associated with substantial one-year mortality, particularly in patients with FFP III fractures [4]. While conservative treatment is generally considered appropriate for stable FFP I injuries, surgical intervention may be more favorable for unstable fracture patterns, especially FFP III. In contrast, the rarity of FFP IV fractures limits the strength of available evidence, and FFP II fractures continue to lack a clear treatment consensus. This uncertainty has prompted increasing interest in surgical strategies that can provide sufficient stability while minimizing invasiveness in frail elderly patients.

Surgical management has advanced significantly, with minimally invasive techniques now the standard for unstable posterior ring injuries [5]. Among surgical options for posterior pelvic ring fixation, iliosacral (IS) screws and transiliac–transsacral (TITS) screws are commonly employed. A retrospective series of elderly patients with osteoporotic posterior pelvic ring fractures reported a significant decrease in pain levels following percutaneous IS screw fixation, with a relatively low rate of intra- and postoperative complications [6]. However, the biomechanical stability of conventional iliosacral screws may be compromised in osteoporotic bone due to limited screw purchase within the sacrum, potentially leading to screw loosening or insufficient fixation in unstable fracture patterns. Screw loosening with back-out is seen in 14–20% of IS screw fixation with 18% necessitating operative revision [7,8,9]. These limitations have prompted the development of alternative fixation strategies aimed at enhancing posterior pelvic ring stability in patients with poor bone quality. TITS screws have gained attention in recent years due to their ability to traverse the entire sacrum and anchor securely in the contralateral ilium. This design provides enhanced biomechanical stability compared to traditional IS screws, reducing micromotion at the fracture site and enabling early mobilization [2,7,10,11].

Despite these advantages, TITS fixation is technically demanding. The narrow osseous corridor requires meticulous preoperative planning and precise intraoperative guidance to avoid complications such as screw loosening, neural injury, or suboptimal fixation [2,12]. Furthermore, limited clinical data on long-term outcomes and potential complications present additional challenges to the broader adoption of this technique.

This study aims to address these gaps by demonstrating the clinical and radiographic outcomes, safety, and feasibility of TITS screw fixation in treating FFP. Our study does not claim superiority but demonstrates that TITS is an option with acceptable outcomes.

## 2. Materials and Methods

### 2.1. Study Design and Sample Population

A retrospective review was conducted on patients who underwent TITS fixation for posterior pelvic ring injuries in Kaohsiung Veterans General Hospital (KVGH) between 2019 and 2024. Institutional Review Board (IRB) approval was obtained prior to the initiation of data collection. Inclusion criteria were patients diagnosed with posterior pelvic ring fragility fractures based on clinical and radiographic assessments (both plain films and CT scans), who underwent TITS screw fixation. Exclusion criteria included high-energy trauma cases, pathological fractures due to malignancy, and incomplete medical records. All clinical factors, including perioperative outcomes (operative time, blood loss, hospital stay, intraoperative complications), radiographic outcomes (reduction quality, screw loosening), and functional outcomes (pain scores using the Visual Analog Scale [VAS], mobility at discharge), were collected through a comprehensive review of electronic medical records (EMR).

### 2.2. Indications for Surgery and Post-Operative Management

Surgical intervention was considered for patients with unstable posterior pelvic ring injuries, including Rommens type III and IV fractures, as well as selected type II fractures with persistent pain, inability to mobilize, or radiographic progression despite conservative management. The decision to operate was made by the treating surgeons based on clinical presentation, imaging findings, and overall patient condition.

The choice of fixation level was determined according to individual sacral anatomy, fracture pattern, and available osseous corridors, as assessed by preoperative computed tomography. Additional anterior pelvic ring fixation was performed at the surgeon’s discretion when anterior instability was identified.

Postoperatively, patients were encouraged to mobilize early as tolerated. Weight-bearing status was individualized based on fracture stability and overall condition. Osteoporosis management was provided according to institutional protocols, and rehabilitation programs focused on early ambulation and functional recovery.

### 2.3. Surgical Technique

Preoperative planning began with identifying the optimal entry point for the TITS screw on a midline sagittal CT reconstruction. This point was then transferred to the lateral pelvic radiograph by referencing cortical landmarks on the sacrum. The feasibility of the selected entry point was assessed by confirming its relationship to key anatomical structures, ensuring it was anterior and superior to the sacral neuroforaminal tunnel, posterior to the anterior sacral cortex, and caudal to the superior sacral endplate.

Intraoperatively, under fluoroscopic guidance, this predetermined entry point was used to insert a guidewire, allowing for controlled directional advancement. Then the cannulated TITS screw was placed across the sacrum from one ilium to the other. Cannulated screws of 7.3 mm diameter were used. Fixation was performed at the S1 and/or S2 level in all patients. This approach aligns with previously described techniques emphasizing the importance of precise imaging magnification, intraosseous trajectory confirmation, and biplanar analysis using standard CT and Picture Archiving and Communication System (PACS) tools [13].

### 2.4. Radiographic Assessment

Postoperative radiographic evaluation was performed using standard anteroposterior pelvic radiographs and follow-up imaging at regular outpatient visits. Radiographs immediately postoperatively, at first follow-up, 1-month, 3-month, 6-month, and final follow-up, were collected. Reduction quality was assessed according to the criteria described by Tornetta and Matta [14,15], which classify alignment as excellent, good, fair, or poor based on residual displacement. Screw loosening was evaluated on follow-up radiographs and classified into two categories. Simple backout was defined as partial screw disengagement without loss of fracture alignment or pelvic ring stability until fracture union. Mechanical failure was defined as screw loosening associated with loss of reduction, instability of the pelvic ring, or the need for revision surgery. These definitions were applied consistently across all cases.

### 2.5. Statistical Analysis

Statistical analyses were performed using SPSS software v29.0.2.0 (IBM Corp., Armonk, NY, USA). Continuous variables were summarized using descriptive statistics. Comparisons between preoperative and postoperative outcomes were performed using the Wilcoxon signed-rank test. A *p*-value of less than 0.05 was considered statistically significant.

## 3. Results

### 3.1. Patient Characteristics

Twenty-two patients were included, all of whom were female. The mean age was 79.0 ± 7.9 years. The average body mass index (BMI) was 23.5 ± 4.6 kg/m^2^, and the mean bone mineral density (T-score) was −3.4 ± 0.8, indicating significant osteoporosis. According to Rommens’ classification, 9 patients had Type II fractures, 9 had Type III fractures, and 4 had Type IV fractures. Patient demographics are summarized in Table 1.

### 3.2. Perioperative Outcomes

The median operative time was 74 min (IQR 55–90), and the median blood loss was 5 mL (IQR 5–18). No intraoperative neurovascular injuries or adverse events were reported. The median hospital stay was 7 days (IQR 5–10). The perioperative outcomes are summarized in Table 2.

### 3.3. Radiographic Outcomes

**Figure 1:**
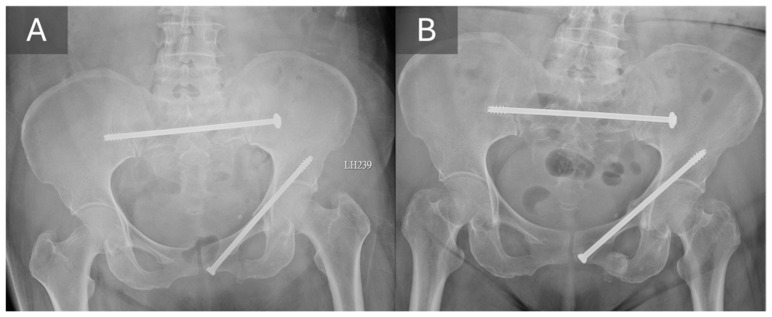
A typical case of using TITS screw in posterior ring fixation. Comparing the radiograph obtained immediate after operation (**A**) with the 6-month follow-up radiograph (**B**), the alignment was well maintained, and no screw loosening was noted.

**Figure 2:**
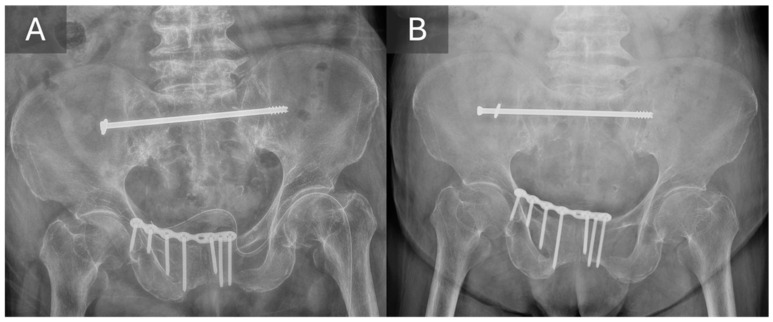
Immediate postoperative radiograph (**A**) and 6-month follow-up radiograph (**B**) from the same patient, demonstrating simple backout of TITS screw without secondary displacement. The alignment of the posterior pelvic ring was maintained.

**Figure 3:**
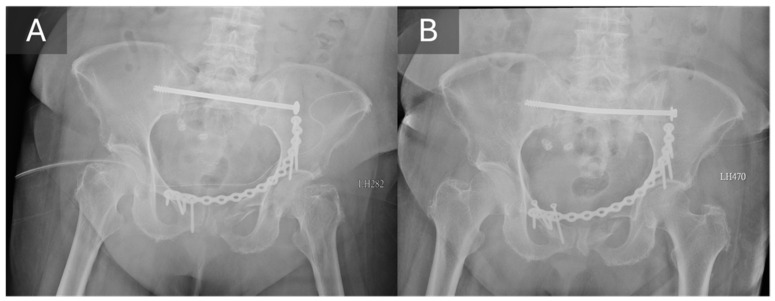
The case with mechanical failure requiring revision surgery in the present study. Though good alignment shown on the radiograph immediate after operation (**A**), TITS screw loosening with back-out and bending was revealed by the 1-month follow-up radiograph (**B**). The back-out of the anterior fixation also indicated instability. Due to the mechanical failure and the persisted pain, the patient underwent a revision surgery.

Eighteen out of 22 patients had maintained satisfying alignment, without screw loosening or secondary displacement of fracture during a mean 6-month follow-up (Figure 1). Screw loosening was observed in four patients (18.2%). We classified loosening of the screw into two categories: simple backout and failure. Simple backout was defined as no fracture re-displacement or progression of screw loosening until union was achieved, although mild disengagement of the screw was present (Figure 2). However, failure was defined as requiring revision fixation, that is, when loosening of the screw influenced pelvic ring stability such that loss of reduction in the fracture or pelvic ring instability was expected. In the present study, there were three simple backouts and one mechanical failure requiring revision surgery (Figure 3). Reduction quality at follow-up was evaluated using criteria from Tornetta and Matta, with 81.8% achieving excellent or good reduction. The radiographic outcomes are summarized in Table 2.

### 3.4. Functional Outcomes

The median VAS score decreased from 5 (IQR 5–6) preoperatively to 2 (IQR 2–3) at discharge (*p* < 0.001). Regarding mobility, no patients were able to ambulate preoperatively during hospitalization. At discharge, 13 patients (59.1%) were able to walk with assistance, while 9 patients (40.9%) still required a wheelchair (*p* < 0.001). Functional outcomes postoperatively were summarized in Table 3.

## 4. Discussion

### 4.1. Outcomes of TITS Fixation

TITS fixation has been shown to be useful in posterior fixation of pelvic ring injuries in several situations, including sacral fractures (predominantly vertically oriented fractures) in young patients, failed IS screw fixation, osteoporotic sacral fractures, minimally displaced SI fracture-dislocation, pelvic malunions/nonunions, and secondary fixation after an IS screw in an unstable fracture pattern [10].

In terms of perioperative outcomes, the median operative time in our study was 74 min, which is longer than that reported by Gewiess et al. (36 min) [16]. This discrepancy may be attributed to the additional anterior fixation performed in some of our patients. Despite the longer operative time, the blood loss remained minimal (median 5 mL), which is comparable to prior studies highlighting the minimally invasive nature of TITS fixation. Moreover, the median hospital stay was 7 days, shorter than the 11 days reported in Gewiess et al.’s cohort, suggesting a benefit in early postoperative recovery.

Regarding radiographic outcomes, 81.8% of our patients achieved excellent or good reduction quality, consistent with previous literature demonstrating favorable alignment outcomes with TITS screws. Our screw loosening rate was 18.2%, aligning with reported rates ranging from 10% to 20% [16,17]. Most loosening cases in our study were simple backouts without clinical consequence, mirroring trends noted in earlier reports.

In functional outcomes, significant improvements were observed. The VAS pain score markedly decreased postoperatively (*p* < 0.001), and 59.1% of patients regained the ability to ambulate with assistance at discharge. These findings are comparable to those of previous studies that reported early mobilization and significant pain relief following TITS fixation [10,16,18]. Our results suggest that TITS fixation not only provides mechanical stability but also facilitates functional recovery, critical in minimizing morbidity in elderly populations.

Taken together, the perioperative, radiographic, and functional outcomes observed in the present study suggest that the clinical value of TITS fixation extends beyond radiographic parameters alone. While maintenance of reduction and implant stability remain important, the ability to achieve early pain relief and mobilization appears to be a more clinically relevant indicator of treatment success in elderly patients with fragility fractures of the pelvis.

In this context, the interpretation of screw loosening deserves particular attention. Although a screw loosening rate of 18.2% may appear concerning at first glance, most cases in our cohort represented simple backout without loss of alignment or functional deterioration. This finding highlights a critical distinction between radiographic changes and clinically meaningful failure in osteoporotic pelvic fixation. In elderly patients with compromised bone quality, minor implant migration may reflect adaptive load redistribution rather than true mechanical instability, especially when posterior ring alignment is preserved and symptoms improve.

These observations have direct implications for surgical decision-making, particularly in fractures that fall outside clearly unstable patterns. While operative fixation is widely accepted for Rommens type III and IV fractures, the management of selected type II fractures remains controversial. In our series, surgical intervention was considered when conservative treatment failed to achieve adequate pain control or mobilization. The favorable functional outcomes observed in these patients suggest that treatment decisions based on functional impairment and mobilization potential, rather than fracture morphology alone, may be appropriate in selected cases.

From a reconstructive standpoint, the biomechanical concept underlying TITS fixation may help explain these clinical findings. By spanning compromised sacral bone and anchoring into both iliac wings, TITS screws redistribute mechanical load across stronger osseous structures and provide robust posterior pelvic stability. This construct may allow early mobilization even in osteoporotic bone, thereby interrupting the cycle of pain, immobility, and medical complications commonly observed in elderly patients with pelvic fragility fractures. A similar biomechanical rationale has been described in previous studies investigating trans-sacral fixation techniques using different implant designs. Wagner et al. reported favorable clinical and functional outcomes following trans-sacral bar osteosynthesis, emphasizing that load transfer through the posterior ilium, rather than reliance on screw purchase within osteoporotic sacral bone, contributes to construct stability and facilitates early mobilization [19]. Although the implant design differs from that of TITS screws, both techniques share the same transsacral fixation concept, aiming to enhance posterior pelvic ring stability by redistributing mechanical forces across stronger cortical bone.

### 4.2. The Importance of Early Ambulation in Osteoporotic Patients

Although conservative management can be considered for FFP type II fractures, which are nondisplaced, all patients in our operative cases are unable to regain their pre-injury levels of mobility and independence. Studies have shown that prolonged bed rest often results in significant morbidity and prolonged hospital stays of 21 to 45 days [20,21]. Breuil et al. observed that 52.5% of elderly patients with osteoporotic pelvic fractures developed complications during hospitalization, with urinary tract infections (50%), bedsores (33%), and cognitive alterations (18%) being the most frequent [22].

Given these concerns, surgical stabilization even for nondisplaced FFP type II fractures has been increasingly discussed as a potential strategy. Among surgical options, TITS fixation stands out due to its minimally invasive nature, biomechanical stability, and ability to support early mobilization. By providing robust stabilization with minimal surgical morbidity, TITS fixation effectively addresses the dual goals of pain control and functional recovery in elderly, osteoporotic patients.

From a practical standpoint, the ability to allow early weight bearing also influences postoperative rehabilitation planning and discharge disposition. Stable posterior pelvic ring fixation may facilitate earlier initiation of physiotherapy, reduce reliance on prolonged bed rest or external support, and potentially shorten the transition to assisted ambulation or home-based care. In clinical practice, these factors are particularly relevant when treating elderly patients with limited social support or pre-existing mobility impairment. Therefore, the benefits of TITS fixation may extend beyond immediate postoperative outcomes to include more streamlined rehabilitation pathways.

### 4.3. Complications

Reported complications associated with TITS fixation include screw loosening, screw malposition, neural injury, implant failure, and infection, with previously published series reporting variable complication rates. In the present study, screw loosening was observed in a subset of patients and was predominantly classified as simple backout without loss of reduction or need for revision surgery, as discussed above.

Importantly, no cases of neural injury, deep infection, or screw malposition were observed in the present cohort. When compared with previously reported series, the incidence of major complications in this study appears comparable or lower, particularly with regard to neurologic and infectious events. These findings suggest that TITS fixation can be performed safely in elderly patients with fragility fractures of the pelvis when appropriate surgical technique and imaging guidance are applied.

### 4.4. Limitations

This study has several limitations that should be acknowledged. First, the retrospective design and relatively small sample size may limit the generalizability of the findings. Second, this was a single-center study, and the results may reflect institution-specific surgical techniques, perioperative management, and rehabilitation protocols, which may differ from those used at other centers.

Third, functional outcomes were assessed primarily during hospitalization and at discharge. While early pain relief and mobilization are clinically meaningful in frail elderly patients, longer-term functional outcomes and patient-reported outcome measures were not systematically evaluated. Additionally, radiographic follow-up was limited to a mean duration of six months, which may be insufficient to fully assess long-term implant survivorship or late complications.

Finally, this study lacked a control group, such as patients treated conservatively or with alternative fixation methods. Therefore, direct comparisons between TITS fixation and other treatment strategies could not be performed. Despite these limitations, the present study provides clinically relevant data on perioperative safety, radiographic outcomes, and early functional recovery following TITS fixation in elderly patients with FFP.

### 4.5. Future Perspectives

Future research should focus on prospective and multicenter studies to further validate the clinical benefits of TITS fixation in fragility fractures of the pelvis. Comparative studies between TITS screws and conventional iliosacral screw fixation may help clarify optimal indications and fixation strategies. Longer follow-up periods are also required to assess implant survivorship, long-term functional outcomes, and the impact of osteoporosis treatment on fixation stability. The integration of surgical stabilization with comprehensive geriatric and osteoporosis management protocols may further improve outcomes in this vulnerable patient population.

## 5. Conclusions

Fragility fractures of the pelvis represent a distinct clinical entity that differs from high-energy pelvic trauma. These fractures frequently occur in frail patients with multiple comorbidities, limited physiological reserve, and reduced capacity to tolerate prolonged immobilization. As a result, treatment strategies must balance mechanical stability with minimal surgical invasiveness. From this perspective, fixation techniques that provide sufficient stability while minimizing surgical trauma are particularly attractive for elderly populations. The present study suggests that transiliac–transsacral screw fixation is a safe and feasible method for posterior pelvic ring stabilization in elderly, osteoporotic patients with fragility fractures of the pelvis. It provides reliable mechanical stability, significant pain relief, promotes early mobilization, and is associated with a short hospital stay, thereby minimizing complications related to prolonged immobility.

## Figures and Tables

**Table 1:** Demographic data of the study population.

Patient Characteristics	
Number	22
Age (years)	79.0 ± 7.9
Gender (F/M)	22/0
BMI (kg/m^2^)	23.5 ± 4.6
T-score	−3.4 ± 0.8
Rommens type	
Type II	9
Type III	9
Type IV	4

**Table 2:** Perioperative and radiographic outcomes.

Variable	Median (IQR) or n (%)
**Perioperative outcomes**	
Operation time (min)	74 (55–90)
Blood loss (mL)	5 (5–18)
Intraoperative adverse events	0
Length of stay (d)	7 (5–10)
**Radiographic outcomes**	
Screw loosening	4 (18.2)
Simple backout	3 (13.6)
Failure	1 (4.5)
Quality of reduction	
Excellent/Good	18 (81.8)
Fair	4 (18.2)
Poor	0 (0)

**Table 3:** Functional outcomes based on the patient condition on discharge.

Variable	Median (IQR) or n (%)		
**Functional outcomes**	**Pre-operative**	**On discharge**	
VAS	5 (5–6)	2 (2–3)	*p* < 0.001
Mobility			*p* < 0.001
Walk with assistance	0 (0)	13 (59.1)	
Wheelchair	22 (100)	9 (40.9)	

## Data Availability

The data presented in this study are available on request from the corresponding author due to privacy and ethical restrictions.
